# Supplementary material for: Photocatalytic decolorization of three commercial dyes using a new heteropolyoxotantalate catalyst
Source: R Soc Open Sci. 2019 Aug 28;6(8):190015. doi: 10.1098/rsos.190015 (PMC6731712; doi:10.1098/rsos.190015)
Supplement: Supporting Information [file rsos190015supp1.docx]

**Supplementary Information**

Photocatalytic decolorization of three commercial dyes using a new heteropolyoxotantalate catalyst

Yansong Wang^a,b^ and Zhimin Zhou^a,^*

*^a^Institute of Natural Resources and Environment, College of Environment and Planning, Henan University, Kaifeng, 475004, China E-mail: pom@henu.edu.cn; Fax: (+86)371-23881850.*

*^b^* *National Demonstration Center for Environmental and Planning, Henan University, Kaifeng, 475004 Henan, China.*

**CONTENTS**

1. **Figure S1. Ball-and-stick representation of P_2_Ta_3_ fragment (a), PO_4_ environment (b) and TaO_7_ coordination environment (c).**
2. **Figure S2. Viewing from different direction of** **ball-and-stick representations of polyanion 1.**
3. **Table S1. Bond lengths in 1a.**
4. **Table S2. BVS calculation results of all the atoms on polyanion 1a.**
5. **Figure S3. Ball-and-stick representation of 1 highlighting the pronated oxygen atoms.**
6. **Figure S4. IR spectrum of 1a in the region between 4000 to 500 cm^−1^.**
7. **Figure S5. XRD patterns of compound 1a.**
8. **Figure S6. Chemical structures of RhB, MB and AR1.**
9. **Figure S7. Photocatalytic decolorization performance of compound 1a without light on RhB dye.**
10. **Figure S8. Photocatalytic decolorization performance of compound 1a on RhB.**
11. **Figure S9. The TOC removal of RhB dye treated by material 1a.**
12. **Figure S10. a) The time-scale UV spectra for the solution of compound 1a; b) the UV spectra of compound 1a and RhB mixed solution before and after irradiation.**
13. **Figure S11. Negative-ion ESI-MS spectra of compound 1a.**
14. **Figure S12. IR spectra of compound 1a before and after catalysis.**
15. **Figure S13. Photocatalytic decolorization performance of compound 2a on RhB dye.**
16. **Figure S14. Photocatalytic decolorization performance of compound 1a on MB.**
17. **Figure S15. Photocatalytic decolorization performance of compound 1a on AR1.**
18. **Figure S16. The photocatalytic H_2_ evolution of compound 1a.**
19. **Figure S17. Schematic illustration for the photocatalytic mechanism of compound 1a.**

**
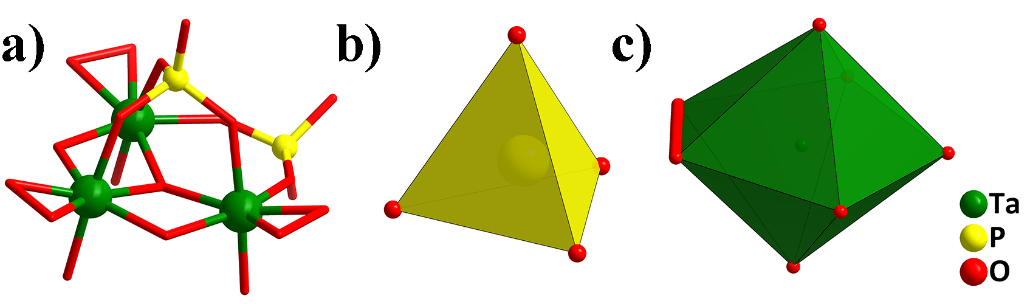
**

**Figure S1. Ball-and-stick representation of P_2_Ta_3_ fragment (a), PO_4_ environment (b) and TaO_7_ coordination environment (c).**

**
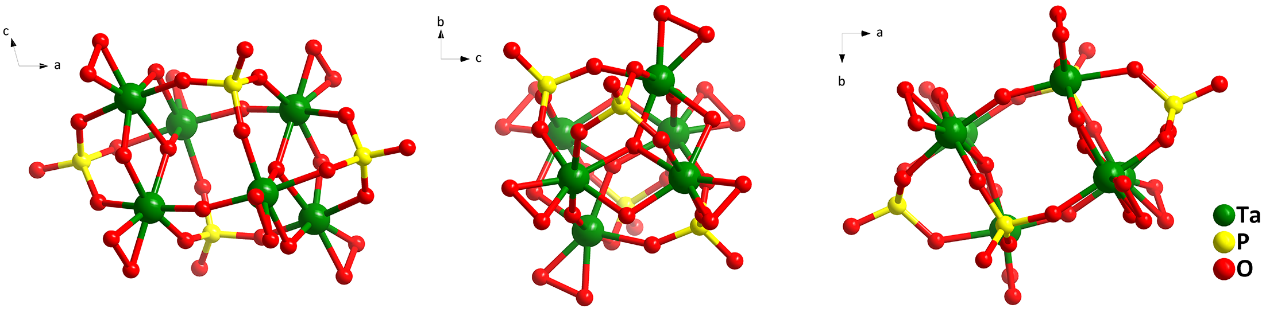
**

**Figure S2. Viewing from different direction of ball-and-stick representations of polyanion 1 (a-c) and the ellipsoid structure of polyanion 1 (d).**

**Table S1. Bond lengths in 1a.**

| Ta–O distance (Å) | | | | | |
| --- | --- | --- | --- | --- | --- |
| Ta1–O1 | 1.918 | Ta2–O4 | 2.046 | Ta3–O1 | 1.916 |
| Ta1–O2 | 1.964 | Ta2–O5 | 2.096 | Ta3–O5 | 2.099 |
| Ta1–O3 | 1.946 | Ta2–O8 | 1.955 | Ta3–O10 | 2.094 |
| Ta1–O4 | 2.070 | Ta2–O9 | 1.946 | Ta3–O13 | 1.973 |
| Ta1–O5 | 2.096 | Ta2–O10 | 2.052 | Ta3–O14 | 1.957 |
| Ta1–O6 | 2.056 | Ta2–O11 | 2.022 | Ta3–O15 | 2.070 |
| Ta1–O7 | 2.047 | Ta2–O12 | 1.984 | Ta3–O17 | 2.027 |
| O–O distance (Å) | | | | | |
| O2–O3 | 1.477 | O8–O9 | 1.509 | O13–O14 | 1.496 |
| P–O distance (Å) | | | | | |
| P1–O6 | 1.549 | P1–O11 | 1.559 | P1–O15 | 1.560 |
| P1–O16 | 1.485 | P2–O7 | 1.528 | P2–O12 | 1.541 |
| P2–O17 | 1.524 | P2–O18 | 1.503 |  |  |

**Table S2. BVS calculation results of all the atoms on polyanion 1a.**

| Atom Code | Bond Valence | Atom Code | Bond Valence | Atom Code | Bond Valence |
| --- | --- | --- | --- | --- | --- |
| Ta1 | 5.52 | O3^Ψ^ | 0.93 | O11 | 1.89 |
| Ta2 | 5.47 | O4* | 1.38 | O12 | 2.02 |
| Ta3 | 5.44 | O5 | 1.86 | O13^Ψ^ | 0.87 |
| P1 | 4.80 | O6 | 1.85 | O14^Ψ^ | 0.90 |
| P2 | 4.97 | O7 | 1.94 | O15 | 1.80 |
|  |  | O8^Ψ^ | 0.91 | O16* | 1.38 |
| O1 | 2.02 | O9^Ψ^ | 0.93 | O17 | 1.99 |
| O2^Ψ^ | 0.89 | O10‡ | 1.32 | O18‡ | 1.32 |

Ψ, ‡ and * represent peroxo oxygen atoms, mono-pronated groups and O/OH ligands, respectively.

**
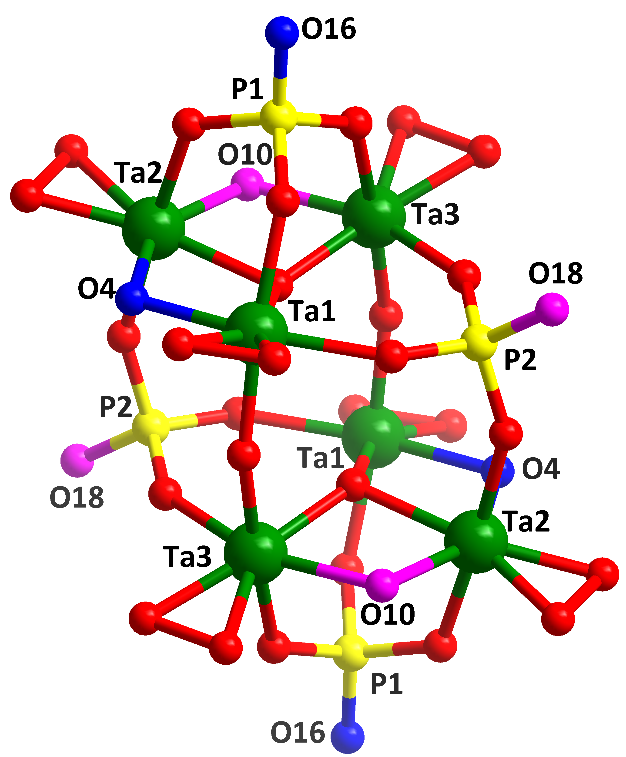
**

**Figure S3. Ball-and-stick representation of 1 highlighting the pronated oxygen atoms. Color code: Ta green balls; P yellow balls, O red balls, OH pink balls, disordered O/OH blue balls.**

**
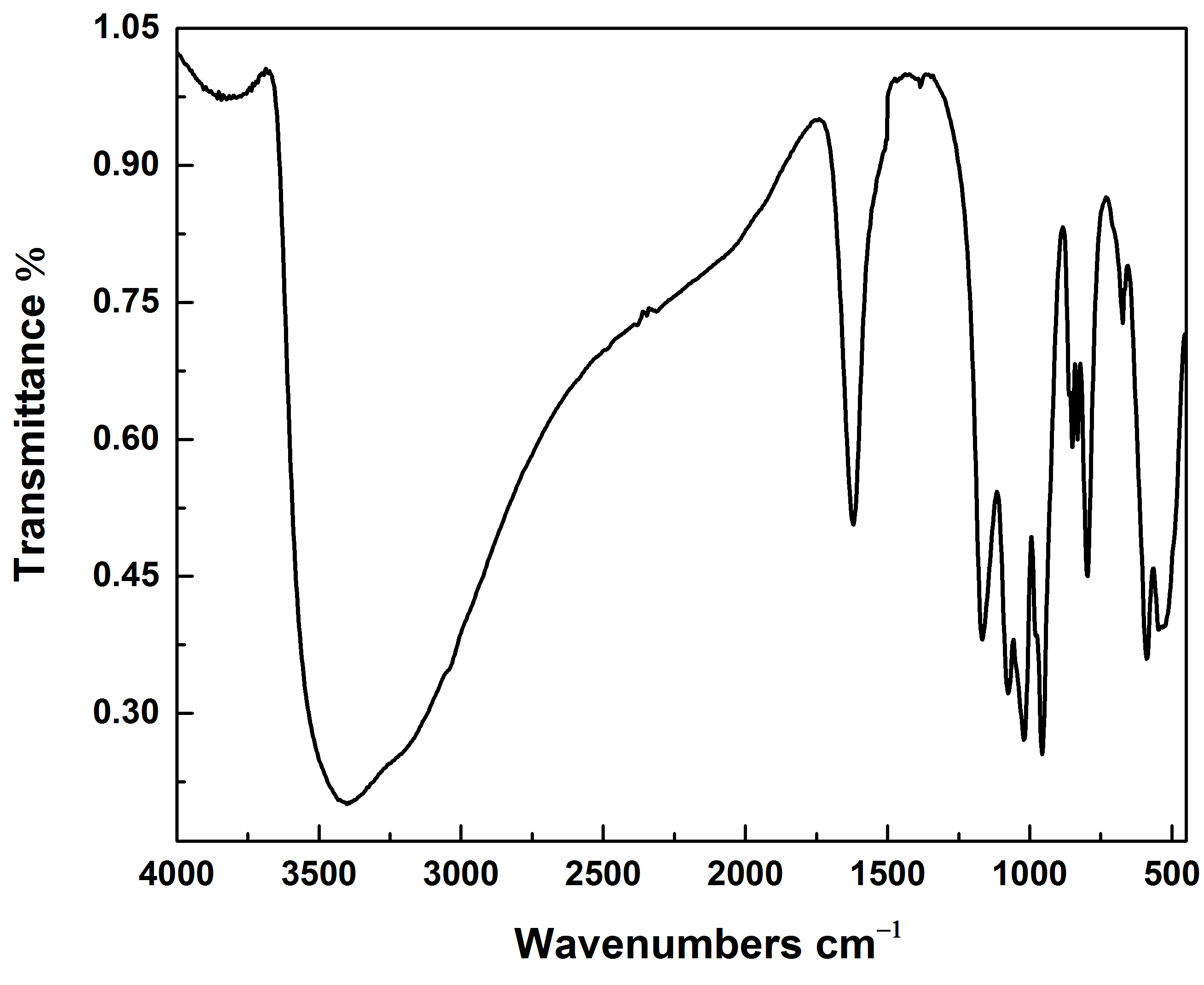
**

**Figure S4. IR spectrum of 1a in the region between 4000 to 500 cm^−1^.**


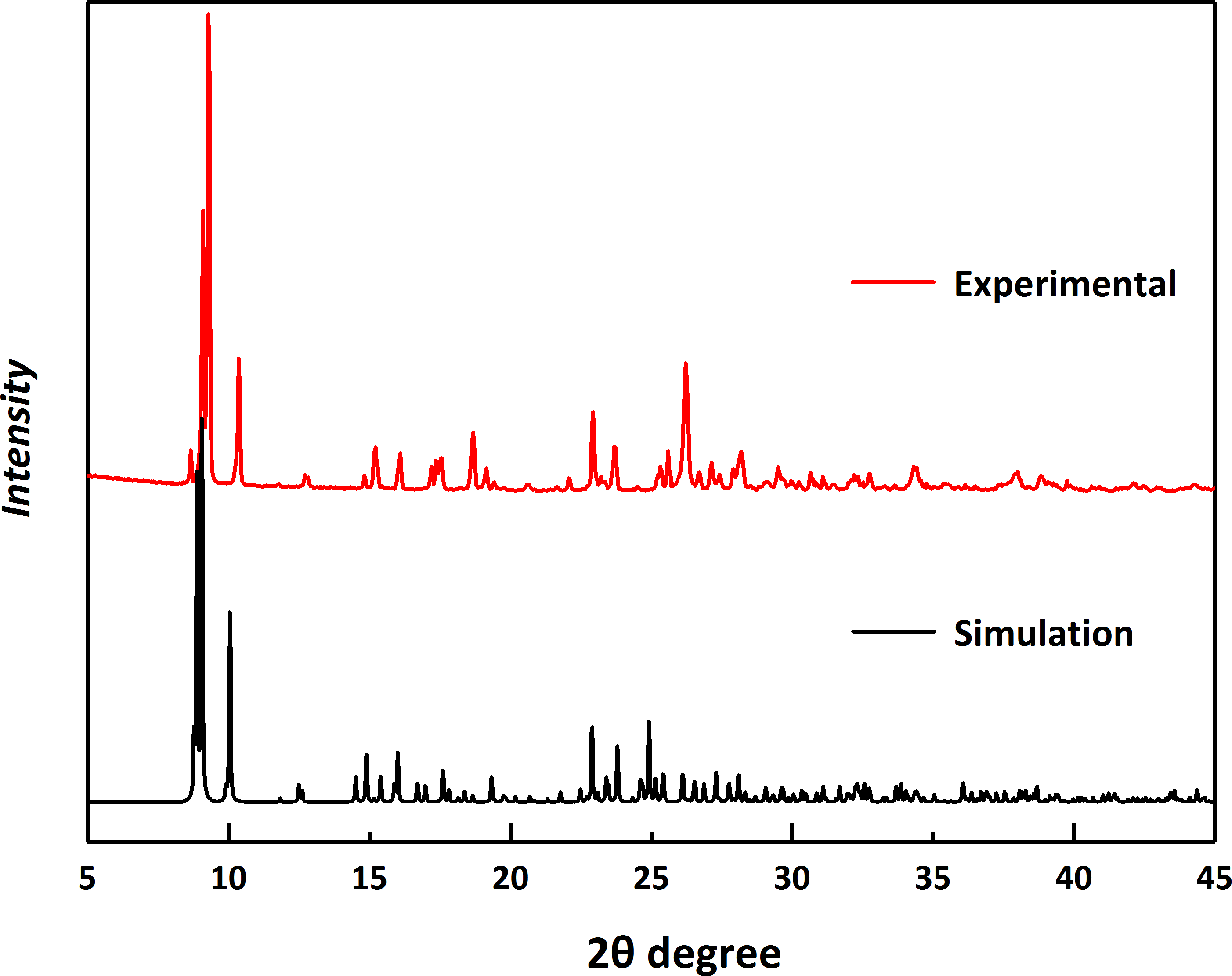


**Figure S5. XRD pattern of compound 1a (red) and its simulated XRD pattern (black).**

**
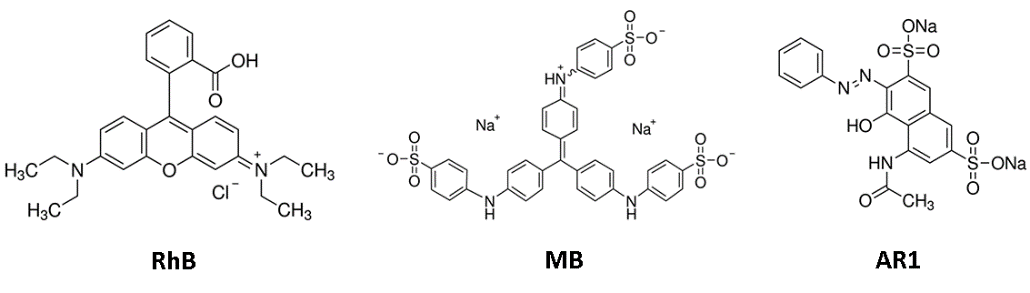
**

**Figure S6. Chemical structures of RhB, MB and AR1.**

**
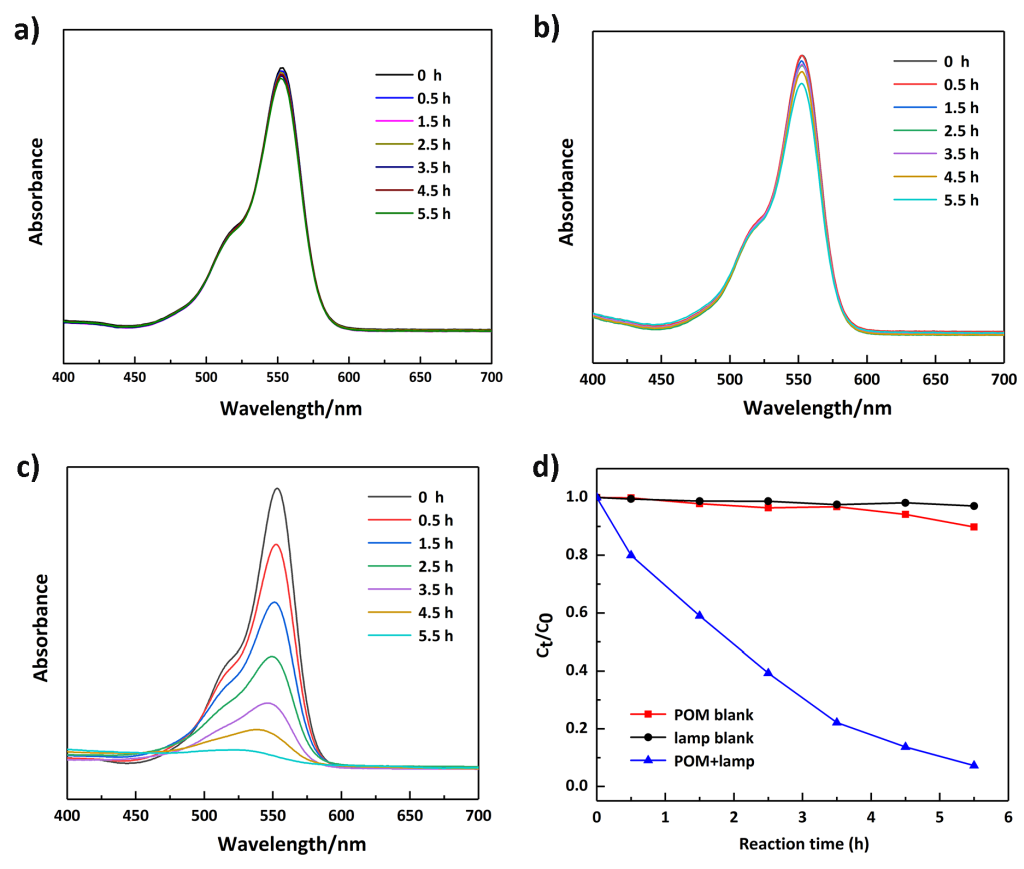
**

**Figure S7.** **Photocatalytic decolorization performance of compound 1a on RhB dye. a) Change in absorption of RhB in the absence of 1a with Xe lamp; b) Change in absorption of RhB in the presence of 1a (40 mg) without Xe lamp; c) Change in absorption of RhB in the presence of 1a (40 mg) with Xe lamp; d) Plot of *C_t_*|*C_0_* vs time with 1a (blue), the absence of 1a (black) and the blank experiment without lamp (red).**

**
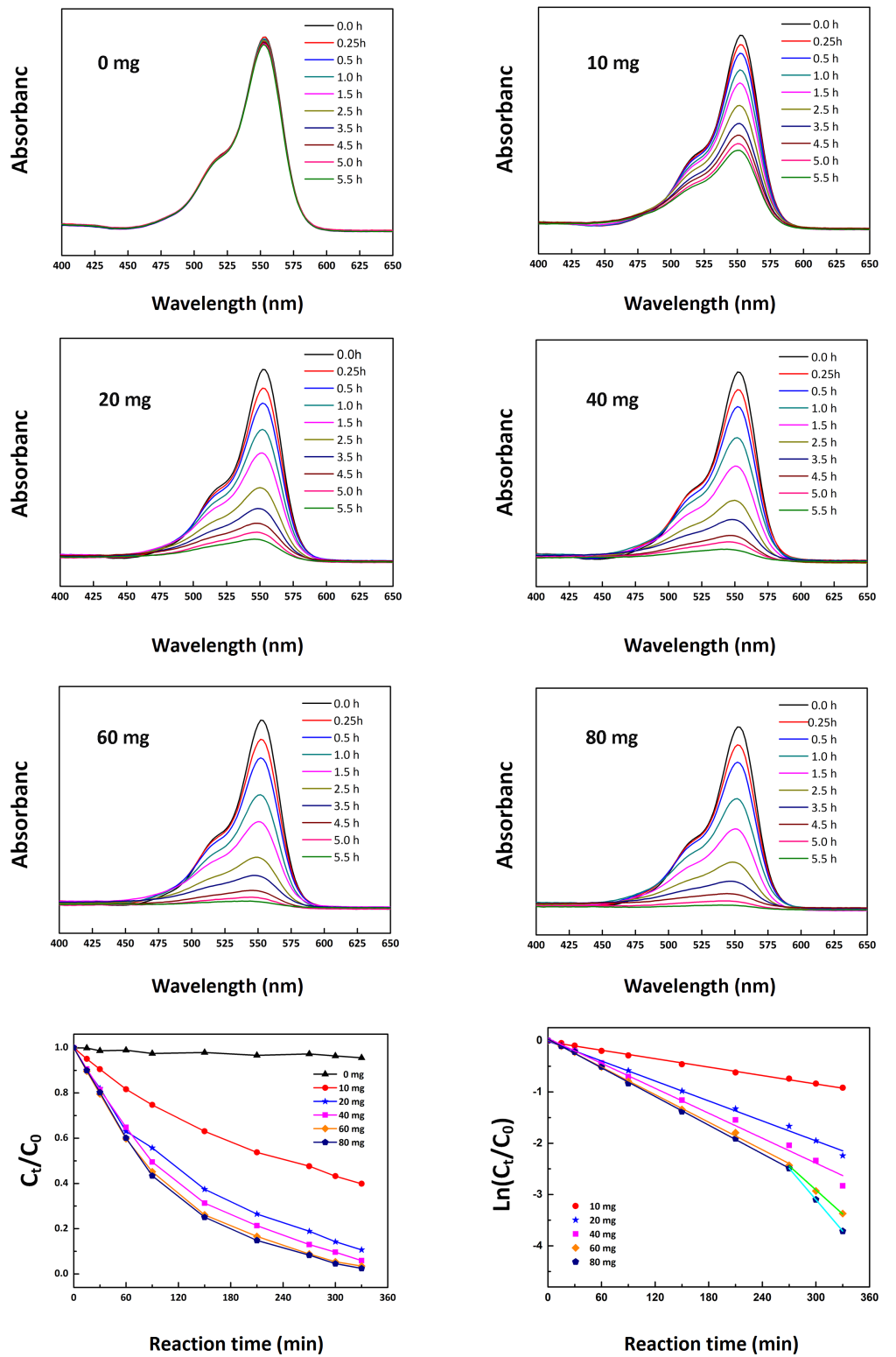
**

**Figure S8. Photocatalytic decolorization performance of compound 1a on RhB dye.**


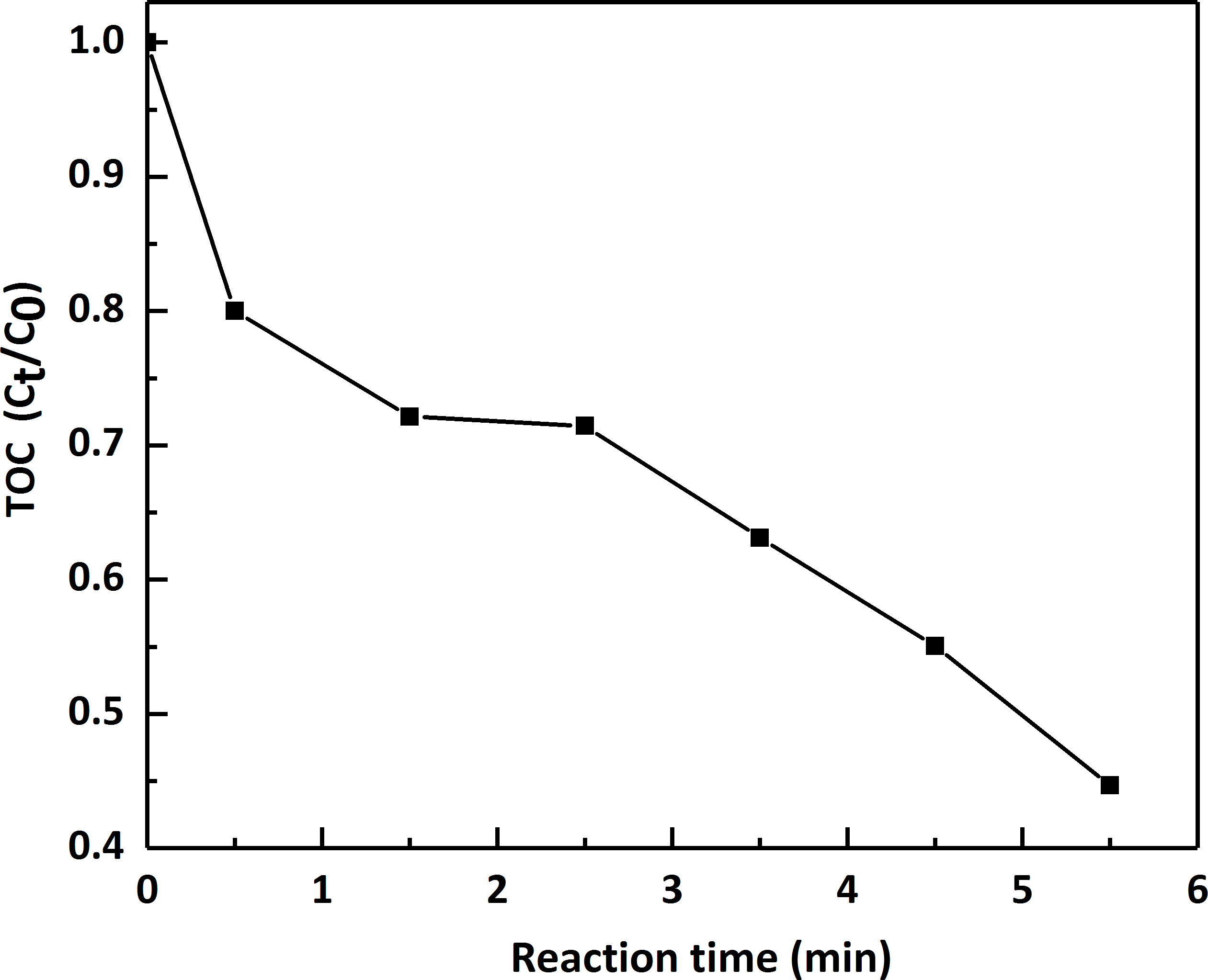


**Figure S9. The TOC removal of RhB dye treated by material 1a.**

**
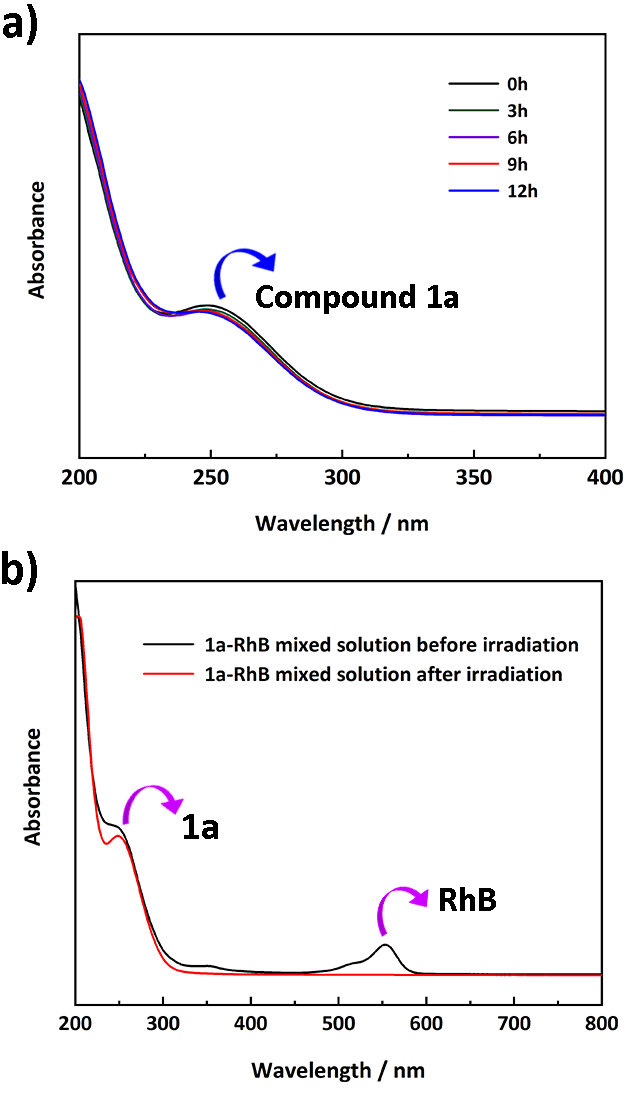
**

**Figure S10. a) The time-scale UV spectra for the solution of compound 1a; b) the UV spectra of compound 1a and RhB mixed solution before and after irradiation.**

**
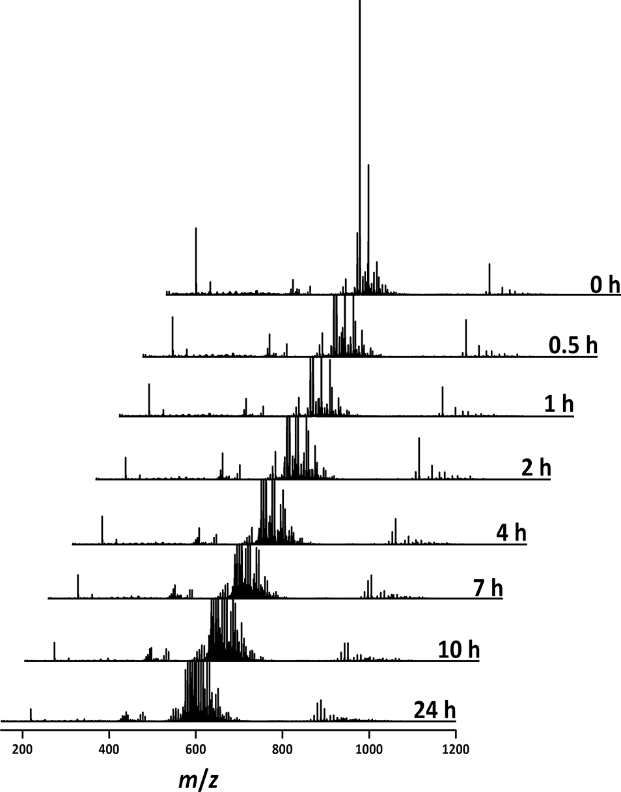
**

**Figure S11.** **Negative-ion ESI-MS spectra of cluster anion in compound 1a during 24 h.** To investigate the stability of POM cluster presented in this study, ESI-MS measurement was performed on an *AB SCIEX Triple TOF 4600* spectrometer operating in negative ion mode and data was analyzed using the *Peakview 2.0* software provided. ESI-MS sample was prepared by dissolving single crystal in mixed solvent CH_3_CN-H_2_O with HPLC grade and diluting to a concentration of ca. 1 x 10^−6^ M. It can be seen that the main envelope observed at *m/z* = 597.5 for **1a** could be assigned to the formula [H_7_{P_4_Ta_6_(O_2_)_6_O_24_}]^3−^, which can be also remained for 24 h.

**
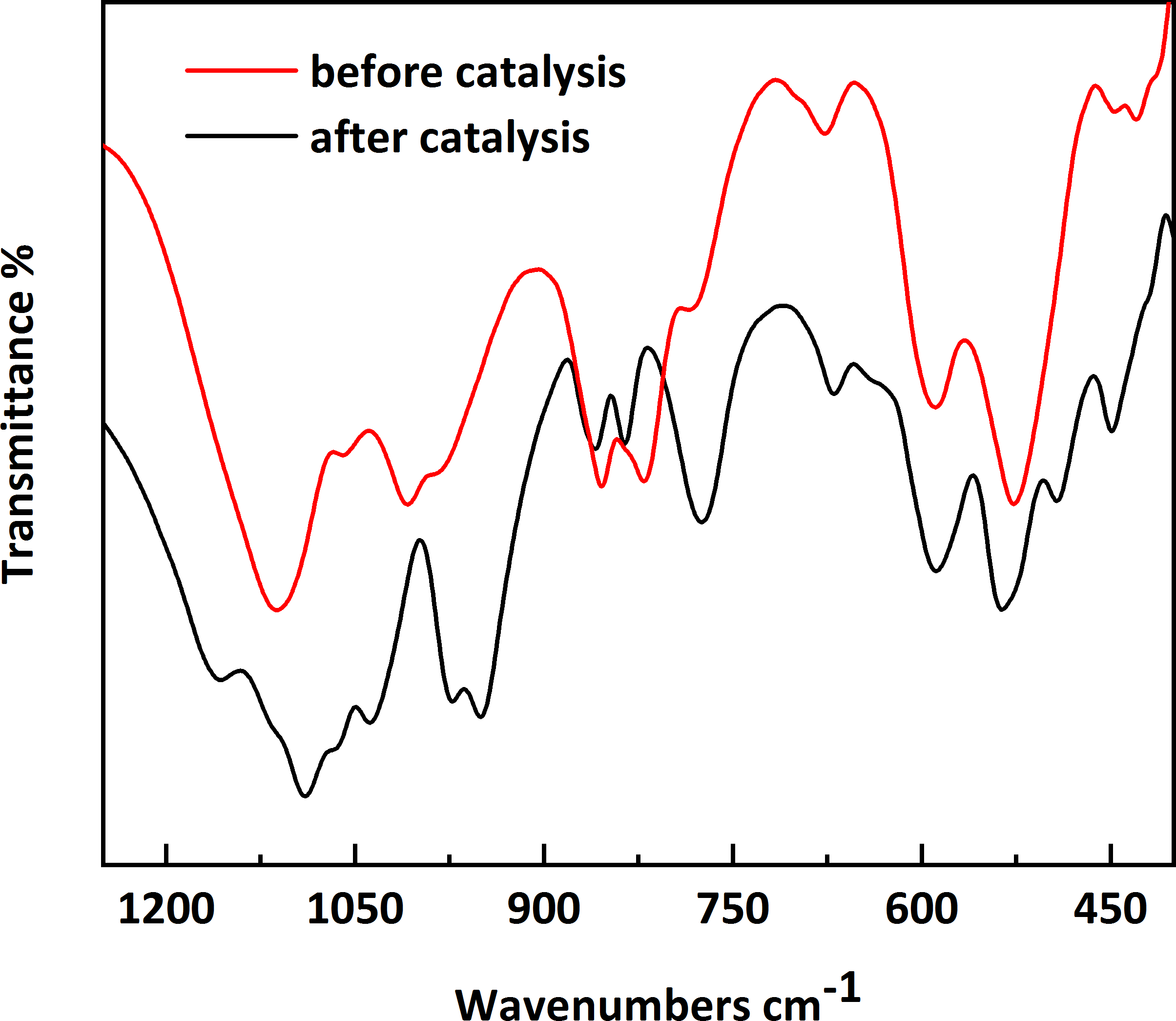
**

**Figure S12. IR spectra of compound 1a before and after catalysis, highlight the region between 1250 to 400 cm^−1^.**

**
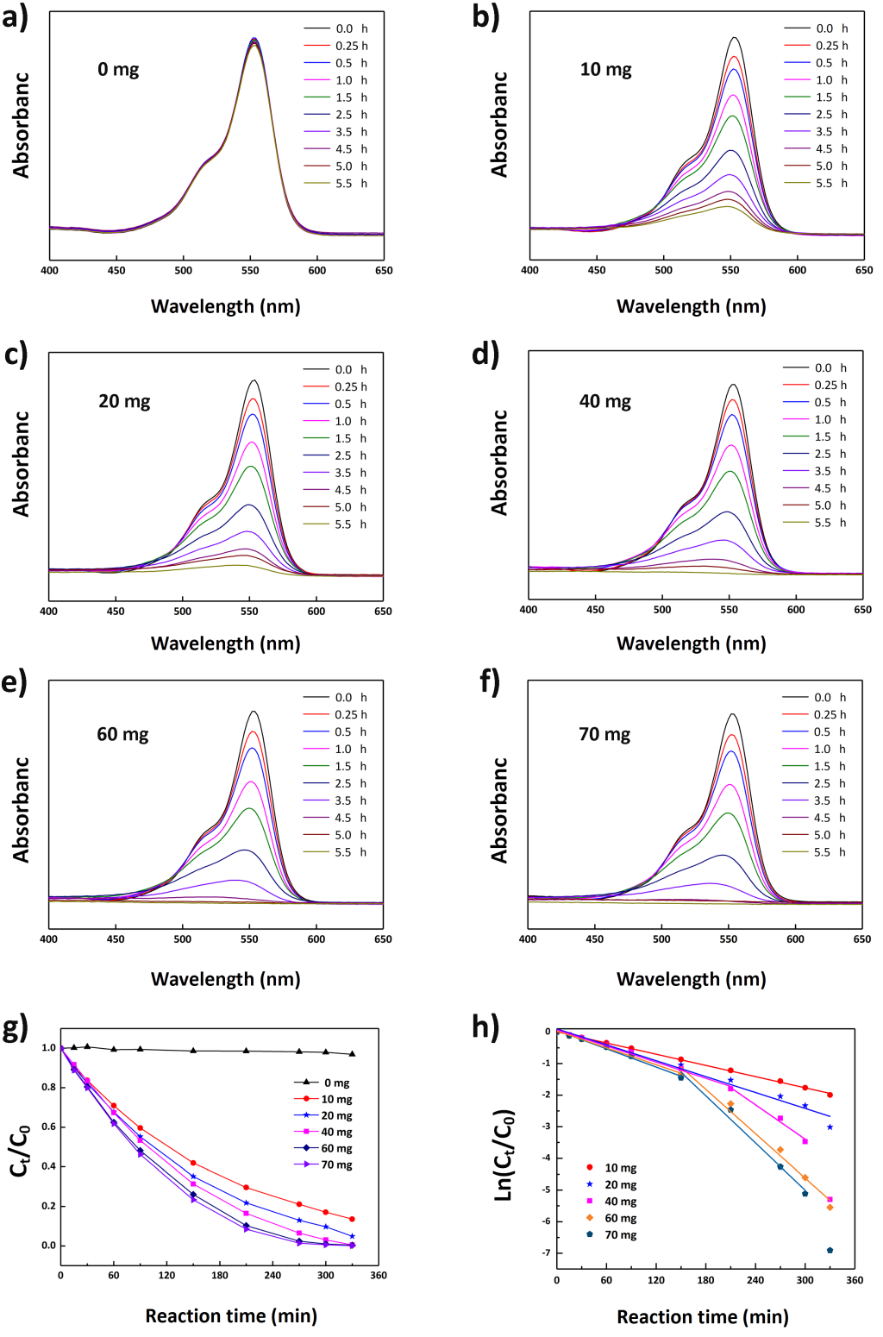
**

**Figure S13. Photocatalytic decolorization performance of compound 2a on RhB dye.**

For comparison, the photocatalytic performances of different amount **2a** (10, 20, 40, 60 and 70 mg) on the decolorization of RhB dyes have been also investigated. It can be seen that RhB dye can be completely decolorized within 5.5 h when the amount of compound **2a** is higher than 20 mg. This may be attributed the fact that **1a** is a structural analogue of **2a**.

**
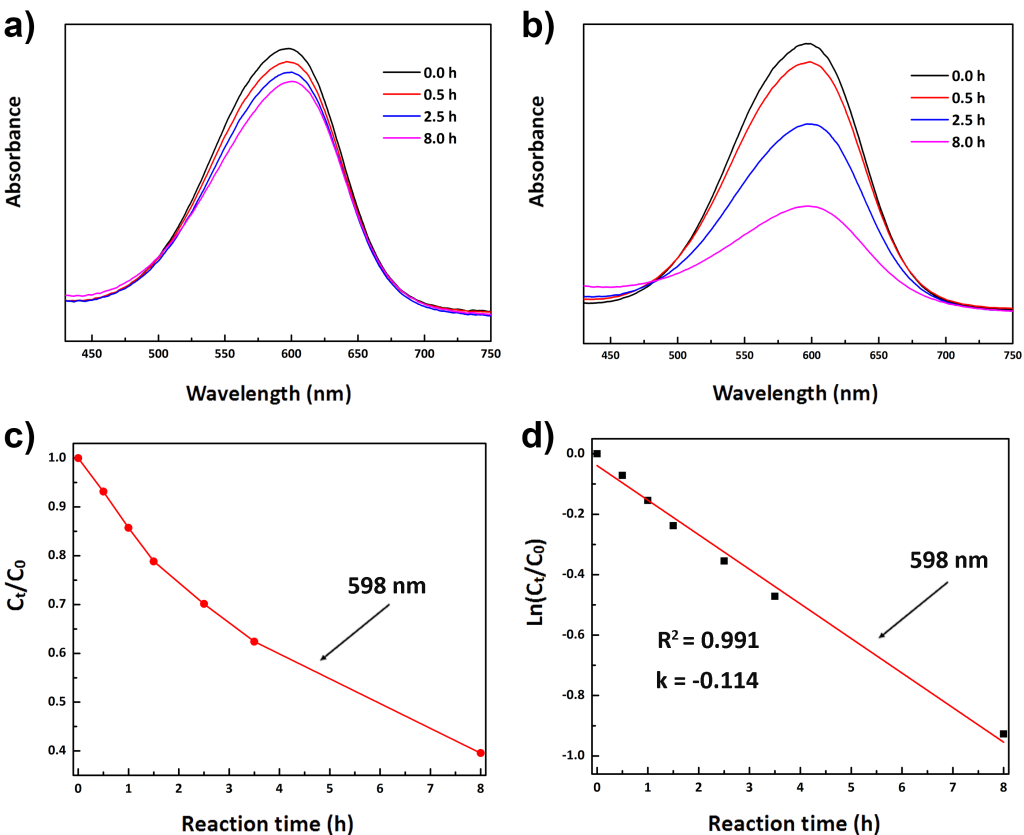
**

**Figure S14. a) Absorption spectrum of the MB solution in the absence of compound 1a; b) Absorption spectrum of the MB solution in the presence of compound 1a (40 mg); c) plots of the concentration ratios of MB *C_t_*|*C_0_* vs time (h) in the presence of compound 1a under Xe lamp irradiation; d) The first order linear plot of *ln*(*C_t_*|*C_0_*) vs. time for MB.**

It can be clearly seen that the absorbance peaks of MB (598 nm) decreased obviously along with the reaction time in the presence of **1a**, 60.4% of MB decolorized after 8 h of irradiation, which is higher than that of absence of **1a** (12.5%). The catalytic reaction could be considered as a pseudo first order kinetics with regard to the linear fit of the *ln*(*C_t_*|*C_0_*) data. The calculated rate constant (*k_app_*, h^–1^) for the reduction of MB in the presence of **1a** is 0.114 h^–1^.

**
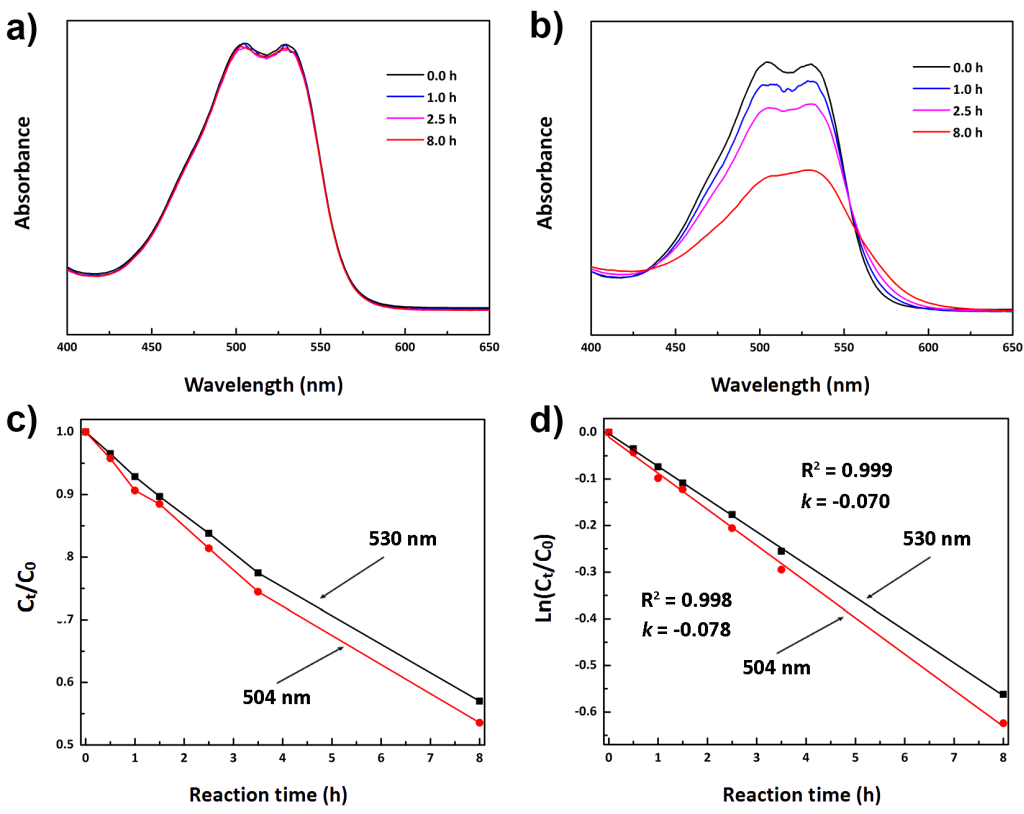
**

**Figure S15. a) Absorption spectrum of the AR1 solution in the absence of compound 1a; b) Absorption spectrum of the AR1 solution in the presence of compound 1a (40 mg); c) plots of the concentration ratios of AR1 *C_t_*|*C_0_* vs time (h) in the presence of compound 1a under Xe lamp irradiation; d) The first order linear plot of *ln*(*C_t_*|*C_0_*) vs. time for AR1.**

It can be clearly seen that the absorbance peaks of AR1 (504 and 530 nm) decreased along with the reaction time in the presence of **1a**, 46.4% (504 nm) and 42.7% (530 nm) of AR1 decolorized after 8 h of irradiation, which is obviously higher than that of absence of **1a** (almost no decolorization). The catalytic reaction could be considered as a pseudo first order kinetics with regard to the linear fit of the *ln*(*C_t_*|*C_0_*) data. The calculated rate constant (*k_app_*, h^–1^) for the reduction of AR1 in the presence of **1a** is 0.078 h^–1^ (504 nm) and 0.070 h^–1^ (504 nm).

**
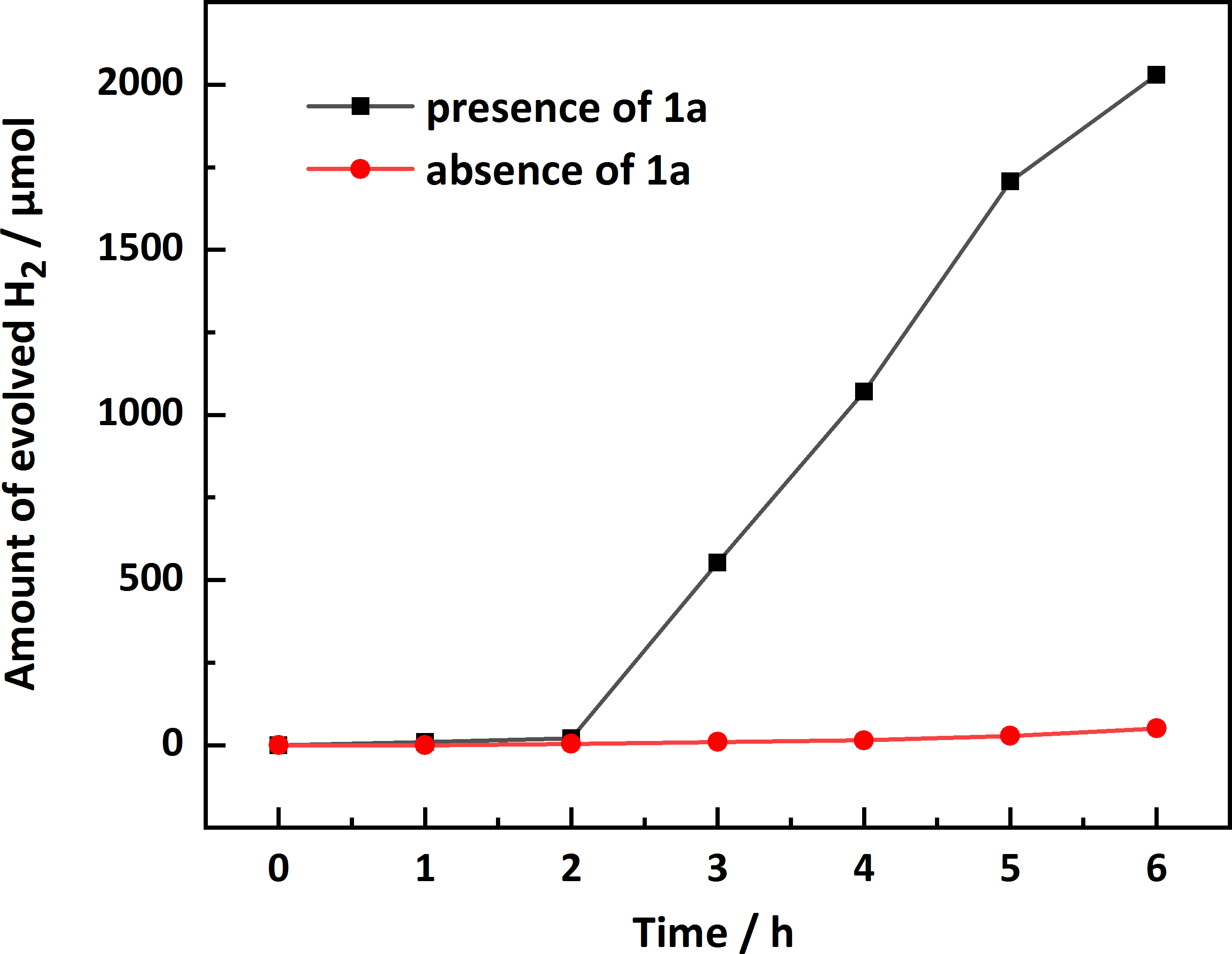
**

**Figure S16. Time courses of photocatalytic H_2_ evolution under the presence of compound 1a (black) and the absence of compound 1a (red). Experiment condition: 100 mg of 1a and 0.05 mg of H_2_PtCl_6_ were dissolved in 100 mL of 10% methanol, which was irradiated under UV using a 500 W mercury lamp.**

**
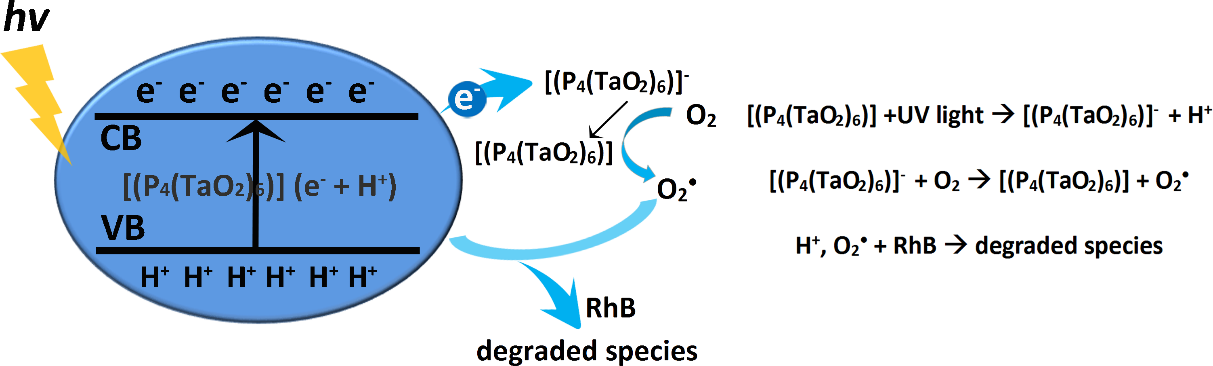
**

**Figure S17. Schematic illustration for the photocatalytic mechanism of compound 1a.**
